# Determination of colistin in luminal and parietal intestinal matrices of chicken by ultra‐high‐performance liquid chromatography‐tandem mass spectrometry

**DOI:** 10.1111/jvp.13022

**Published:** 2021-10-06

**Authors:** Andrew Mead, Nathalie Gillard, Christelle Robert, Gilles Pierret, Jean Henrottin, Pascal Richez, Ludovic Pelligand

**Affiliations:** ^1^ The Royal Veterinary College Hatfield UK; ^2^ CER Groupe Marloie Belgium; ^3^ Transpharm Saint‐Genies des Mourgues France

**Keywords:** colistin, digesta, pharmacokinetics, polymyxin, poultry, UHPLC‐MS/MS

## Abstract

Justification for continued use of colistin in veterinary medicine, for example medicated water, relies on pharmacokinetic/pharmacodynamic (PK/PD) studies that require accurate measurement of colistin content in the digestive tract. A method for the detection and quantification of colistin in poultry intestinal material was developed and validated. Colistin is not absorbed after oral administration, and the biophase is the gastrointestinal tract. Extraction of colistin from the matrix was achieved using solid‐phase extraction with a methanol:water (1:2; v/v) solution. Polymyxin B was used as an internal standard. Colistin A and colistin B, the main components of colistin, were separated, detected and measured using ultra‐high‐performance liquid chromatography coupled with tandem mass spectrometry (UHPLC‐MS/MS). The method was validated for linearity/quadraticity between 1.1 (LOQ) and 56.7 mg/kg. Mean accuracy was between 82.7% and 107.7% with inter‐ and intra‐day precision lower than 13.3% and 15% respectively. Freeze–thaw, long‐term and bench storage were validated. Incurred samples following colistin treatment in poultry at the approved clinical dose of 75,000 IU/kg in drinking water and oral gavage were quantifiable and in line with expected intestinal transit times. The method is considered appropriately accurate and precise for the purposes of pharmacokinetic analysis in the gastrointestinal tract.

## 1 INTRODUCTION

Colistin is a critically important antimicrobial for last‐line treatment of multi‐drug‐resistant Gram‐negative infections in humans (WHO, 2017), but also for treating gastrointestinal infections in livestock, including poultry (Apostolakos & Piccirillo, 2018; Kempf et al., 2016; Poirel et al., 2017; Rhouma et al., 2016). Colistin use in animals varies between countries, with reports indicating high use in Asia, Europe and South America (Kempf et al., 2013; Webb et al., 2017). The discovery of mobilized colistin resistance elements from *E*. *coli* in pigs raises concerns that use in livestock production may accelerate resistance selection and dissemination in animals and humans (Liu et al., 2016; Shen et al., 2016; Walsh & Wu, 2016).

Safeguarding colistin as a last‐line antibiotic requires enhanced understanding of digestive pharmacokinetics (PK) in livestock, specifically the transit of colistin through the luminal intestinal content (LIC), residual binding to parietal intestinal content (PIC) and delayed excretion due to differential rates of emptying in luminal caecal content (LCC) and parietal caecal content (PCC) (Clench & Mathias, 1995; Guyonnet et al., 2010).

Colistin contains multiple compounds: predominantly colistin A (polymyxin E_1_) and colistin B (polymyxin E_2_), the proportions of which vary dependent on supplier and batch, making quantification difficult (Brink et al., 2014). Previous methods include microbiological assays (Sato et al., 1972), immunological assays (Kitagawa et al., 1985) and, more recently, high‐performance liquid chromatography (HPLC) coupled with tandem mass spectrometry (MS/MS) (Cangemi et al., 2016; Chepyala et al., 2015; Fu et al., 2018). These methods quantified colistin in human plasma following intravenous administration, which may not be suitable for more complex matrices, like gastrointestinal (GI) content. Measurement of colistin in plasma is negligible following oral administration and would not provide a valid quantification of the antimicrobial impact in the GI tract. Here, we describe extraction of colistin from poultry intestinal matrices, luminal and parietal content, using SPE purification and measurement via UHPLC‐MS/MS to quantify colistin for pharmacokinetic/pharmacodynamic (PK/PD) investigations.

European Pharmacopoeia compliant Meiji Seika Pharma's Colistin sulphate (ColiMeiji^®^, hereafter ‘colistin’) consisting of 78.53% of colistin A (polymyxin E_1_) and colistin B (polymyxin E_2_) was supplied by Wyjolab. Stock standard solutions of colistin and polymyxin B (internal standard) were prepared at 1000 µg/ml (colistin base equivalent) by dissolving the compounds in water and storing at 4°C, with fresh stocks prepared daily, considering the purity and water content.

A novel UHPLC‐MS/MS method was validated for the measurement of colistin in the intestinal matrix, as extracted from the small intestine of chickens. Validation was performed using nontreated luminal intestinal content spiked with appropriate volumes of standard stock colistin solution and internal standard. Matrix‐matched QC samples between 1.1 and 28.4 mg/kg were compared with a calibration curve covering 1.1–56.7 mg/kg colistin base. For sample extraction, 500 µl of 2% bovine‐serum albumin (BSA) was added to each sample (200 mg), vortexed and incubated at room temperature for 10 min to limit the adsorption of colistin binding to plastic and improve recovery. 1.5 ml of extraction solution, methanol: 4 M sulphuric acid (1:2; v/v), was added and mechanically agitated for 30 min, and optimized for protein precipitation and maximal colistin recovery (Fu et al., 2018). After centrifugation, colistin was isolated from the supernatant via solid‐phase extraction and eluted in methanol:formic acid (99.9:0.1; v/v), dried at 50°C under nitrogen stream and reconstituted in 200 µl water:formic acid (99.9:0.1; v/v). Chromatographic analyses were performed on an Acquity ultra‐performance liquid chromatography system with a BEH C_18_ separation column (1.7 µm particle size, 2.1 × 50 mm) (Waters). The column and autosampler were maintained, respectively, at 50°C and 10°C, and the injection volume was 20 µl. Mobile phases consisted of 0.1% formic acid in water (solvent A) and 0.1% formic acid in acetonitrile (solvent B). The flow rate and solvent gradient varied according to Table S1.

The UHPLC system was coupled to a Xevo TQS‐Micro triple quadrupole mass spectrometer (Waters). The mass spectrometer was operated with positive electrospray ionization (ESI) in multiple reaction monitoring (MRM) mode (full method in supplementary materials, Table S2). Colistin concentration was calculated as the ratio of the sum of peak areas of colistin A and B over the internal standard polymyxin B_1_ peak area.

A total of 112 samples were analysed using the validated method: 100 LIC, four PIC, four LCC and four PCC. Samples were collected *postmortem* from chickens dosed (between 13 and 16 days old) with colistin sulphate, via drinking water or oral gavage at the approved clinical dose of 75,000 IU/kg. Birds were fed baby chick crumbs (Small holder Range), a feed free of coccidiostats, designed to feed from hatching to 6–8 weeks. A matrix‐matched calibration curve was prepared with each batch of samples for quantification. This study was approved by the local ethical committee and completed in accordance with ASPA (1986) legislation (PPL number: PCCBD6D98).

Calibration curves were obtained by least‐squares quadratic regression with a weighting factor (1/x²) and excluding the origin. The correlation coefficients *R*² of the calibration curves were above 0.99 for the 5 validation days, and regression was assessed by ANOVA (Table S3). Specificity was acceptable, with negligible carry over of 0.02 mg/kg, far below LOQ (1.1 mg/kg). Accuracy and precision at the LOQ, within run (RSDr) and between‐run (RSDR) precision and accuracy were acceptable (Tables S4 and S5). Colistin spiked samples showed acceptable stability at 4°C, long‐term frozen storage, stability during analysis and multiple freeze/thaw cycles, indicating that storage up to fifteen weeks was achievable and that freeze/thawing had no significant impact on recovery (Tables S6–S8).

**FIGURE 1:**
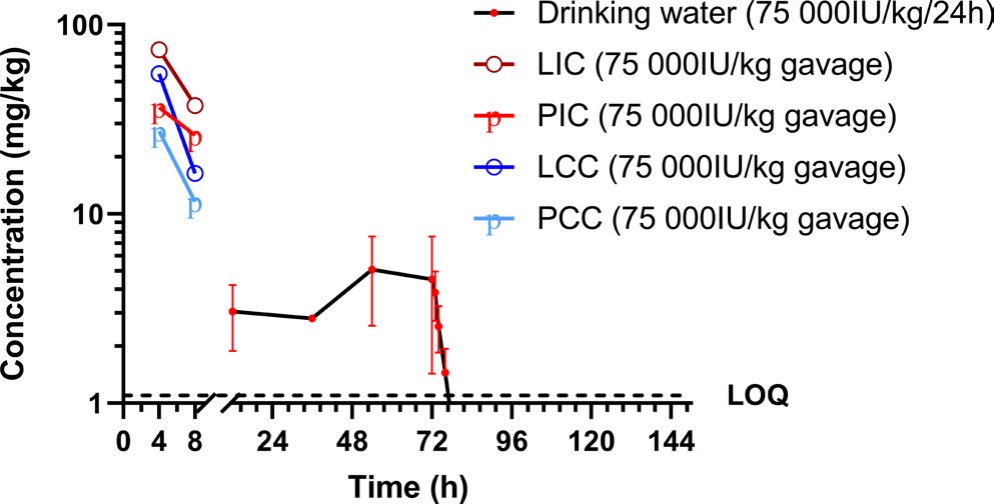
Measurement of colistin in incurred samples of luminal intestinal content (LIC), parietal intestinal content (PIC), luminal caecal content (LCC) and parietal caecal content (PCC) from chickens dosed via drinking water and oral gavage at 75,000 IU/kg

Quantification of total colistin from samples collected during and after dosing is shown in Figure 1 (Tables S9 and S10). A gradual increase in LIC during dosing was observed, followed by a rapid decline in line with expected transit time for poultry digesta, along with measurable concentrations in PIC, LCC and PCC. This demonstrates method suitability for the purposes of colistin quantification for pharmacokinetics in complex intestinal matrices relevant to its clinical use for enteric treatment.

Accurate measurement of colistin concentration within the digestive tract is key for pharmacokinetics, devising accurate and effective dosing profiles, and making policy decisions. Colistin presents several challenges for HPLC; high binding affinity is problematic for sample preparation and column loading, and it lacks native fluorescence and presents a weak UV signal. It is composed of many compounds, making individual compound separation difficult, although methods utilizing the combination of HPLC with tandem mass spectrometry have accurately separated and quantified polymyxin E_1_ and E_2_ (Gobin et al., 2010, van den Meersche et al., 2016). Methods quantifying colistin demonstrate LOQs of 38.1 µg/L in urine (Zhao et al., 2016) 68.9 µg/kg in muscle tissue (Boison et al., 2015), and 117.3 µg/kg in manure (van den Meersche et al., 2016). The higher limits reported here are attributed matrix complexity. Batch matching colistin for analytical and animal phases provides that the MS response and colistin ratio are equivalent, regardless of proportion. The sum of polymyxin E_1_ and E_2_ ensures accurate quantification when reporting a digestive PK profile although low antimicrobial effects of other components represent a limitation of the UHPLC‐MS/MS method when compared with microbiological methods utilizing antimicrobial effect (Guyonnet et al., 2010; Sato et al., 1972). Although UHPLC‐MS/MS methods provide more precise measurements, the preprocess purification and deproteination results in total colistin measurements, which require further analysis of the protein binding fraction to account for ‘free’ and unbound colistin.

Although absorption of colistin is negligible, impact of protein binding/binding to materials within the digesta may limit ‘free’ colistin, the subsequent antimicrobial efficacy, and how the dose is related to the pharmacokinetics in the GI tract. Guyonnet et al. (2010) demonstrated that for pig gut liquor, the apparent ratio between antimicrobial effect and colistin as measured by HPLC was 0.8:1. However, this may be different in chicken intestinal matrix due to differences in digesta, which cannot be accounted for without further study. Varying constitution of the intestinal matrix, due to dietary conditions, may impact on the accuracy of this method. A secondary study (not reported here) successfully used this method to quantify colistin in LIC with older birds (35 days old) fed a grower feed (complete flour‐based feed), but further validation is needed to explore the robustness of this technique between different feeding profiles and laboratories.

Results from samples tested here show that the method is suitable to quantify colistin for developing a digestive PK profile. Compared with the profile published by Sato et al. (1972), which showed high concentrations within the small intestine at eight hours, our study shows a more rapid elimination, with colistin levels below the LOQ within four hours of dosing cessation. This is likely related to physiological differences in gut transit time between the 6‐month‐old layer hens and 16‐day‐old broiler chicks, and impacted by methodological differences between reporting total colistin via UHPLC‐MS/MS and ‘free’ colistin using a microbiological method.

Determination of colistin pharmacokinetics is vital for designing efficacious treatments. This paper describes a UHPLC‐MS/MS method that is specific, accurate, precise and suitable for quantifying colistin in chicken intestinal matrices. Its limit of quantification was validated at 1.1 mg/kg, corresponding to the lower end of typical MIC values for pathogenic *E*. *coli*. This method is suitable for optimizing PK data and future PK/PD predictions and informing colistin usage.

## CONFLICT OF INTEREST

The authors declare that they have no known competing financial interests or personal relationships that could have appeared to influence the work reported in this paper.

## ANIMAL WELFARE AND ETHICS STATEMENT

This study was reviewed and approved by Royal Veterinary College ethics and welfare committee in accordance with ASPA (1986) legislation (PPL number: PCCBD6D98).

## Supporting information

Supplementary Material

## Data Availability

The data that support the findings of this study are available in the supplementary material of this article.
