# Supplementary material for: Determination of colistin in luminal and parietal intestinal matrices of chicken by ultra‐high‐performance liquid chromatography‐tandem mass spectrometry
Source: J Vet Pharmacol Ther. 2021 Oct 6;44(6):982–5. doi: 10.1111/jvp.13022 (PMC9293311; doi:10.1111/jvp.13022)
Supplement: Supplementary file 1 — Supplementary Material [file JVP-44-982-s001.docx]

Supplementary

***Reagents***

UHPLC-grade water, acetonitrile, methanol and formic acid were acquired from Biosolve (Valkenswaard, The Netherlands). Sulfuric acid and bovine serum albumin (BSA) were obtained from Sigma-Aldrich (Bornem, Belgium). Oasis HLB solid-phase extraction cartridges (6 mL, 200 mg; Waters, Milford, MA, USA). Nitrogen gas was supplied by Air Liquide (Milmort, Belgium). Polymyxin B was used as an internal standard, and was provided by LGC Standards (Molsheim, Germany).

***Sample preparation***

Luminal material was collected from intestines from non-treated chicken at slaughterhouse, homogenized and 200 mg (± 2 mg) aliquots transferred to 15 mL disposable centrifuge tubes for preparation of test samples. These blank test samples were spiked with colistin and polymyxin B (internal standard), through the addition of appropriate volumes of working solution, to prepare calibration and quality control (QC) samples. A matrix-matched calibration curve was prepared at the following levels 1.1, 1.7, 2.3, 5.7, 11.3, 22.7, 39.7, and 56.7 mg/kg and QC samples were 1.1, 2.8, 14.2, and 28.4 mg/kg.

For sample extraction, 500 µL of 2% BSA was added to each sample (200 mg), vortexed and incubated at room temperature for 10 minutes. 1.5 mL of extraction solution consisting of methanol: 4M sulphuric acid (1:2; v/v) was added and the mixture was shaken in a mechanical agitator for 30 minutes. Centrifugation at 4660 g for 5 minutes at 4°C was followed by the decanting of the supernatant and addition of 4 mL deionised water. The solution was transferred onto the Oasis HLB column, which was preconditioned with 5 mL of methanol and rinsed with two washes of 4 mL deionised water. The compounds were eluted with 3 mL of methanol:formic acid (99.9:0.1; v/v) in to 15 mL centrifuge tubes and evaporated at 50°C to dryness under nitrogen stream. The dried extract was reconstituted in 200 µL of water:formic acid (99.9:0.1; v/v) and centrifuged at 11750 g for 5 minutes at 20°C. Supernatant was transferred to appropriate autosampler vial prior to instrumental analysis.

***Instrumental conditions***

Chromatographic analyses were performed on an Acquity ultra-performance liquid chromatography system with BEH C_18_ separation column (1.7 µm particle size, 2.1 x 50 mm) (Waters, Milford, MA, USA). The column and autosampler were maintained, respectively, at 50°C and 10°C and the injection volume was 20 µL. Mobile phases consisted of 0.1% formic acid in water (solvent A) and 0.1% formic acid in acetonitrile (solvent B). The flow rate and solvent gradient varied according to table S1.

Supplementary Table 1 (S1): Variable flow rate and gradient for solvents A (0.1% formic acid in water) and B (0.1% formic acid in acetonitrile) used in UHPLC analyte separation.

| **Time (min.)** | **Flow rate (mL/min)** | **Solvent A (%)** | **Solvent B (%)** |
| --- | --- | --- | --- |
| 0 | 0.2 | 90 | 10 |
| 0.2 | 0.2 | 90 | 10 |
| 2.4 | 0.2 | 5 | 95 |
| 2.6 | 0.5 | 0 | 100 |
| 3.6 | 0.5 | 0 | 100 |
| 3.7 | 0.5 | 90 | 10 |
| 4.5 | 0.5 | 90 | 10 |
| 4.7 | 0.2 | 90 | 10 |
| 5 | 0.2 | 90 | 10 |

The UHPLC system was coupled to a Xevo TQS-Micro triple quadrupole mass spectrometer (Waters, Milford, MA, USA). The mass spectrometer was operated with positive electrospray ionization (ESI) in Multiple reaction monitoring (MRM) mode. The ESI parameters were adjusted as follows: source temperature of 150°C, desolvation temperature of 500°C, desolvation gas flow rate 1200 L/h, cone gas flow rate 50 L/h, and cone voltage 15 V. Collision-induced dissociation was done with argon as the collision gas at 4 x 10^-3^ mbar pressure in the collision cell.

Colistin A and B can form multiple charged ions in ionization process. As the triply charged ions had better ion response than doubly charged ions, the triply charged ions were used as the precursors in Multiple reaction monitoring analysis. Parent ions, corresponding daughter ions, and collision energy are reported in table 2. Data acquisition was performed using MassLynx^®^ v4.2 (Waters, Milford, MA, USA).

Supplementary table 2 (S2): Collision energy and corresponding transitions for colistin A, B, and polymyxin B.

| **Compound** | **Parent ion (m/z)** | **Daughter ion/s (m/z)** | **Collision energy (eV)** |
| --- | --- | --- | --- |
| Colistin A  (polymyxin E_1_) | 390.9 | 385 | 8 |
|  |  | 379 | 10 |
|  |  | 101 | 15 |
| Colistin B  (polymyxin E_2_) | 386.1 | 380.2 | 8 |
|  |  | 374.4 | 10 |
|  |  | 101 | 15 |
| Polymyxin B_1_ | 402.1 | 101 | 7 |

***Method validation***

The method was validated by a set of parameters. As the aim of this study is to establish PK/PD criteria for colistin, not to be used for safety assessment of residues, broader acceptance criteria than VICH GL 49 may be accepted (-30/+20% for accuracy and 15 and 20% for within-run and between-run precision, respectively).

The UHPLC-MS/MS method for the determination of colistin was validated for linearity/quadraticity between 1.1 and 56.7 mg/kg of colistin, limit of quantification (LOQ), within- and between-run accuracy and precision, specificity, freeze/thaw stability, extended frozen stability and autosampler stability. Colistin concentration was calculated as the ratio of the sum of peak areas of colistin A and B over the internal standard polymyxin B_1_ peak area (Equation 1).

Equation 1: Calculation of spiked or incurred colistin concentration using peak areas of colistin A, colistin B, and polymyxin B_1_.

$$\frac{Colistin A+Colistin B}{Polymyxin B_{1}}=f(Spiked colistin concentration or matrix equivalent)$$

For checking the linearity/quadraticity calibration standards at 1.1, 1.7, 2.3, 2.8, 5.7, 11.3, 22.7, 39.7, and 56.7 mg/kg were analysed each day for 5 days.

The LOQ is the smallest measured content of colistin at and above which the determination can be made with an accuracy between 70-120 % and a precision expressed as standard deviation [RSD] ≤ 15 %.

Specificity was validated through the analysis and comparison of blank matrix samples (n=3) compared with samples fortified with the lowest calibration standard (n=3); specificity was determined by no significant interfering substances eluting at the same retention time as markers (Supplementary figure 1).

Accuracy, precision, freeze/thaw stability (three cycles), extended frozen stability (7, 11, and 15 weeks) and autosampler stability (up to 48 h at 4°C) were evaluated through recovery at four concentrations (1.1, 2.8, 14.2, and 28.4 mg/kg). Evaluation of precision (repeatability and within-lab reproducibility) and accuracy was represented by respectively RSD (%) and recovery (%) on within run (6 replicates) and between run (3 days). The impact of three repeated freeze/thaw cycles was investigated with samples (n=3) frozen at -70°C for 24 hours and thawed at room temperature for each cycle. Extended frozen stability was checked by storing spiked samples (n=3) at -70°C for 7, 11 or 15 weeks before analysis. Autosampler stability at 2-8°C was shown using sample extracts (n=3) stored at 2-8°C for minimum 24 hours prior to analysis by UHPLC-MS/MS. Validity of these parameters was accepted if it fell within the acceptance criteria for accuracy, i.e. recovery between 70-120 % and a relative standard deviation (RSD) of ≤ 15 %. Details of validation format in appendix A.

Supplementary Table 3 (S3): Calibration results for colistin in poultry intestinal content (n = 10, 1.1 – 56.7 mg/kg)

| **Day** | **Equation** | **Coefficient of determination (R^2^)** |
| --- | --- | --- |
| 1 | y= -0.000565813x^2+0.581046x+0.0458955 | 0.999 |
| 2 | y= 0.00122811x^2+0.520915x+0.155291 | 0.993 |
| 3 | y= 0.000135216x^2+0.690619x+0.179899 | 0.995 |
| 4 | y= 0.00126644x^2+0.478117x+0.29007 | 0.990 |
| 5 | y= -0.00141384x^2+0.629982x+0.0620455 | 0.994 |

Supplementary Table 4 (S4): Accuracy and precision for colistin at LOQ (1.1 mg/kg) and at a higher level of 1.7 mg/kg.

| **QC sample number** | **Colistin concentration (mg/kg)** | |
| --- | --- | --- |
|  | **1.1** | **1.7** |
| 1 | 1.2 | 1.8 |
| 2 | 1.1 | 1.8 |
| 3 | 1.2 | 1.7 |
| 4 | 1.2 | 1.7 |
| 5 | 1.1 | 1.8 |
| 6 | 1.2 | 1.8 |
| Intra-day mean concentration (mg/kg) | 1.2 | 1.8 |
| Intra-day RSD (%) | 3.3 | 1.9 |
| Intra-day RSD (%) Tolerance | 15.0 | |
| Recovery (%) | 101.5 | 103.3 |
| Recovery limits (%) | 70-120 | |

Supplementary Table 5 (S5): Accuracy and precision results for colistin measured in poultry intestinal content.

|  | **Theoretical colistin concentration** | | | | | | | | | | | |
| --- | --- | --- | --- | --- | --- | --- | --- | --- | --- | --- | --- | --- |
|  | **1.1 mg/kg** | | | **2.8 mg/kg** | | | **14.2 mg/kg** | | | **28.4 mg/kg** | | |
|  | **Day 1** | **Day 2** | **Day 3** | **Day 1** | **Day 2** | **Day 3** | **Day 1** | **Day 2** | **Day 3** | **Day 1** | **Day 2** | **Day 3** |
| **QC sample number** | Detected colistin concentration (mg/kg) | | | | | | | | | | | |
| 1 | 1.2 | 0.9 | 1.2 | 3.0 | 3.4 | 2.8 | 12.0 | 15.1 | 11.7 | 22.2 | 25.7 | 25.5 |
| 2 | 1.1 | 1.2 | 0.8 | 2.9 | 3.3 | 2.6 | 12.8 | 15.3 | 12.0 | 23.3 | 27.0 | 25.9 |
| 3 | 1.2 | 1.0 | 1.2 | 2.9 | 3.2 | 2.7 | 12.1 | 15.4 | 11.7 | 22.5 | 30.4 | 25.9 |
| 4 | 1.2 | 1.2 | 1.2 | 2.8 | 3.0 | 2.9 | 12.2 | 13.6 | 12.4 | 21.5 | 25.6 | 24.5 |
| 5 | 1.1 | 1.2 | 1.2 | 2.8 | 2.9 | 2.5 | 11.8 | 15.1 | 13.1 | 21.3 | 30.4 | 22.9 |
| 6 | 1.2 | 1.2 | 1.2 | 2.9 | 2.7 | 2.4 | 10.8 | 14.2 | 11.9 | 21.9 | 33.5 | 24.3 |
| Intra-day mean concentration (mg/kg) | 1.2 | 1.1 | 1.1 | 2.9 | 3.1 | 2.7 | 12.0 | 14.8 | 12.2 | 22.1 | 28.7 | 24.8 |
| Intra-day RSD (%) | 3.3 | 12.3 | 15.0 | 2.2 | 7.9 | 6.6 | 5.5 | 4.9 | 4.5 | 3.3 | 11.0 | 4.7 |
| Intra-day RSD Tolerance (%) | 15 | | | | | | | | | | | |
| Inter-day mean concentration (mg/kg) | 1.1 | | | 2.9 | | | 13.0 | | | 25.2 | | |
| Inter-day RSD (%) | 10.7 | | | 8.6 | | | 11.2 | | | 13.3 | | |
| Inter-day RSD Tolerance (%) | 23 | | | | | | | | | | | |
| Recovery (%) | 100.2 | | | 101.3 | | | 91.3 | | | 88.9 | | |
| Recovery limits (%) | 70-120 | | | | | | | | | | | |

Supplementary *Table 6 (S6): Extended frozen (-70°C) stability of spiked colistin poultry intestinal content samples*

| **QC sample number** | **Theoretical Colistin concentration 1.1 mg/kg** | | | **Theoretical Colistin concentration 2.8 mg/kg** | | | **Theoretical Colistin concentration 14.2 mg/kg** | | | **Theoretical Colistin concentration 28.4 mg/kg** | | |
| --- | --- | --- | --- | --- | --- | --- | --- | --- | --- | --- | --- | --- |
|  | **7 weeks** | **11 weeks** | **15 weeks** | **7 weeks** | **11 weeks** | **15 weeks** | **7 weeks** | **11 weeks** | **15 weeks** | **7 weeks** | **11 weeks** | **15 weeks** |
| 1 | 1.3 | 0.9 | 1.3 | 2.5 | 3.5 | 2.6 | 10.2 | 15.1 | 12.8 | 23.9 | 22.2 | 22.7 |
| 2 | 1.2 | 0.8 | 1.1 | 2.4 | 2.9 | 2.8 | 9.7 | 14.6 | 10.2 | 21.6 | 31.1 | 23.2 |
| 3 | 1.0 | 0.9 | 1.1 | 2.0 | 3.1 | 2.3 | 12.3 | 10.2 | 10.4 | 21.4 | 30.1 | 21.2 |
| Recovery (%) | 102 | 77.1 | 101.4 | 82.2 | 111.9 | 89.4 | 75.5 | 93.8 | 78.5 | 78.5 | 97.9 | 78.7 |
| Recovery limits (%) | 70-120 | | | | | | | | | | | |

| **QC sample number** | **Theoretical Colistin concentration 1.1 mg/kg** | **Theoretical Colistin concentration 2.8 mg/kg** | **Theoretical Colistin concentration 14.2 mg/kg** | **Theoretical Colistin concentration 28.4 mg/kg** |
| --- | --- | --- | --- | --- |
| 1 | 0.8 | 2.6 | 11.2 | 22.1 |
| 2 | 1.2 | 2.6 | 12.9 | 24.4 |
| 3 | 0.9 | 2.7 | 12.1 | 26.8 |
| Recovery (%) | 83.2 | 92.9 | 85 | 85.9 |
| Recovery limits (%) | 70-120 | | | |

Supplementary *Table 7 (S7): Autosampler stability of colistin in poultry intestinal content (storage of sample extracts at 4°C 48 hours)*

Supplementary *Table 8 (S8): Froze/thaw stability of colistin in poultry intestinal content sample*

| **QC sample number** | **Theoretical Colistin concentration 1.1 mg/kg** | | | **Theoretical Colistin concentration 2.8 mg/kg** | | | **Theoretical Colistin concentration 14.2 mg/kg** | | | **Theoretical Colistin concentration 28.4 mg/kg** | | |
| --- | --- | --- | --- | --- | --- | --- | --- | --- | --- | --- | --- | --- |
|  | **1 cycle** | **2 cycles** | **3 cycles** | **1 cycle** | **2 cycles** | **3 cycles** | **1 cycle** | **2 cycles** | **3 cycles** | **1 cycle** | **2 cycles** | **3 cycles** |
| 1 | 1.0 | 1.2 | 1.2 | 2.8 | 2.6 | 3.1 | 14.9 | 14.5 | 15.4 | 26.7 | 30.4 | 25.5 |
| 2 | 0.9 | 1.1 | 1.1 | 2.6 | 2.7 | 2.8 | 15.9 | 14.1 | 14.8 | 29.7 | 26.7 | 30.3 |
| 3 | 0.9 | 1.2 | 0.8 | 2.7 | 3.0 | 3.1 | 15.1 | 13.6 | 15.3 | 27.8 | 28.5 | 26.2 |
| Recovery (%) | 82.7 | 102.4 | 91.9 | 95.8 | 97.9 | 104.7 | 107.7 | 99.2 | 107.0 | 98.9 | 100.5 | 96.2 |
| Recovery limits (%) | 70-120 | | | | | | | | | | | |

Supplementary Figure 1: Chromatogram of a sample fortified at 50% of LOQ (0.57 mg/kg) colistin (left) and blank intestinal content (right) representing MRM of colistin (Colistin A, top; Colistin B, middle) and internal standard (polymyxin B_1_, bottom).


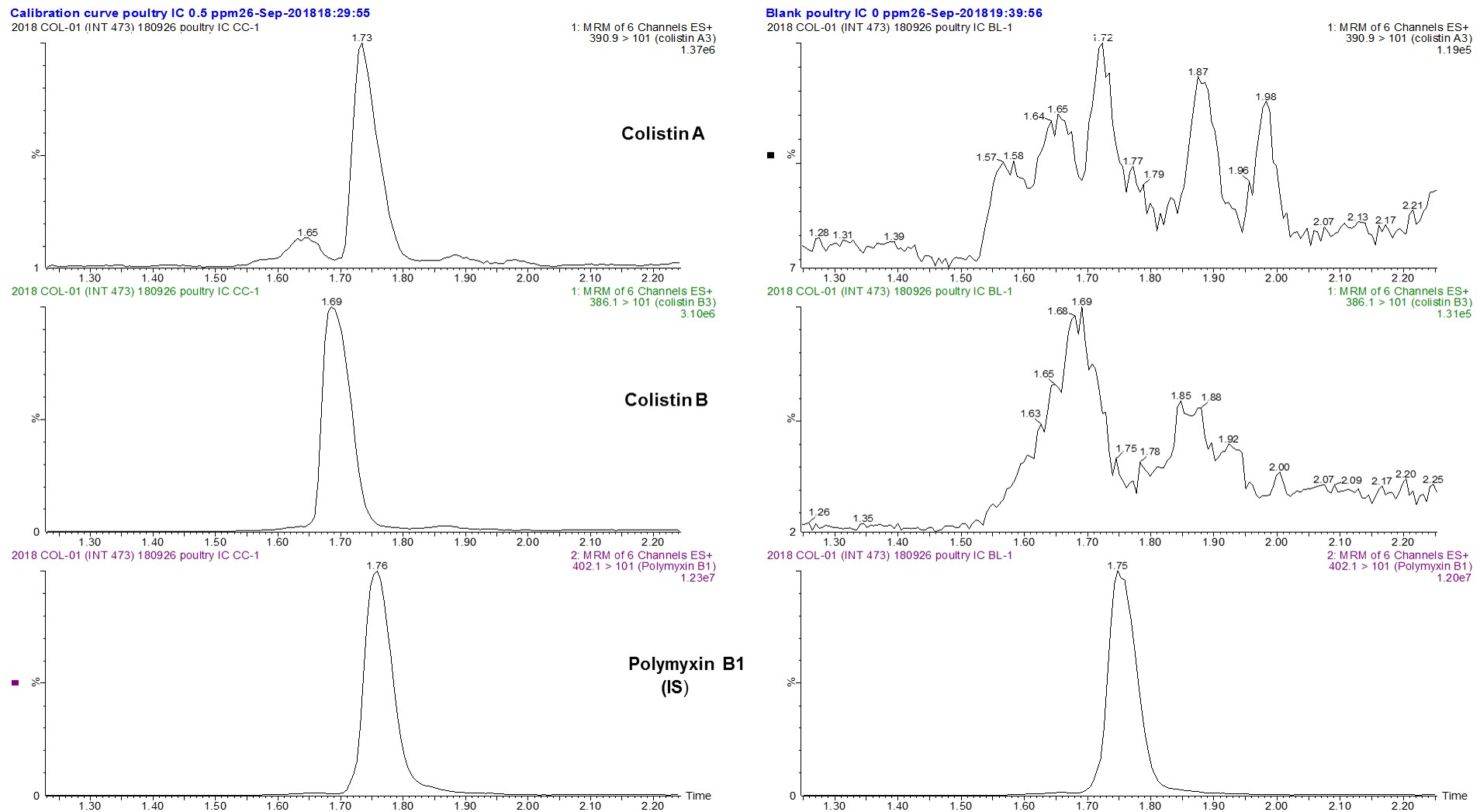


***Incurred samples***

Supplementary Table 9 (S9): Mean colistin concentration measured in luminal intestinal content following 3-day administration of colistin sulphate at 75 000 IU/kg via drinking water, n = 1 to 6 birds per timepoint.

| **Sample time from onset of dosing (h)** | **Mean colistin concentration (mg/kg)** |
| --- | --- |
| 12 | 3.05 |
| 36 | 2.80 |
| 54 | 5.09 |
| 72 | 4.52 |
| 73 | 3.85 |
| 74 | 2.55 |
| 76 | 1.45 |
| 80 | 0.44 |
| 84 | 0.30 |
| 96 | 0.18 |
| 120 | 0.15 |
| 144 | 0.17 |

Supplementary Table 10 (S10): Mean colistin concentration in LIC, PIC, LCC and PCC following oral gavage at clinical dose of 75 000 IU/kg, n = 2 birds per timepoint.

| **Sample time from onset of dosing (h)** | **Mean colistin concentration (mg/kg)** |
| --- | --- |
| **LIC** | |
| 4 | 73.7 |
| 8 | 37.3 |
| **PIC** | |
| 4 | 36.5 |
| 8 | 26.1 |
| **LCC** | |
| 4 | 55.1 |
| 8 | 16.4 |
| **PCC** | |
| 4 | 27.4 |
| 8 | 11.6 |

*LIC – Luminal intestinal content, PIC – Parietal intestinal content, LCC – Luminal caecal content, PCC – Parietal caecal content.

**Appendix A**

Validation scheme for colistin determination in intestinal matrices. Quality control (QC) and intestinal content (IC)

| **Test Sample/s** | **Validation objective** |
| --- | --- |
| Calibration (0.57, 1.13, 1.70; 2.27; 2.84, 5.7, 11.3, 22.7, 39.7, 56.7 mg/kg in IC)  3 blank – IC  6 QC 1.13 mg/kg - IC  6 QC 1.70 mg/kg - IC  6 QC 2.84 mg/kg - IC  6 QC 14.2 mg/kg - IC  6 QC 28.4 mg/kg - IC | Linearity, LOQ determination  (IC)  Accuracy, precision,  specificity (IC) |
| Calibration (0.57, 1.13, 1.70; 2.27; 2.84, 5.7, 11.3, 22.7, 39.7, 56.7 mg/kg in IC)  6 QC 1.13 mg/kg - IC  6 QC 2.84 mg/kg - IC  6 QC 14.2 mg/kg - IC  6 QC 28.4 mg/kg – IC | Linearity,  Accuracy, precision, (IC) |
| Calibration (0.57, 1.13, 1.70; 2.27; 2.84, 5.7, 11.3, 22.7, 39.7, 56.7 mg/kg in IC)  6 QC 1.13 mg/kg - IC  6 QC 2.84 mg/kg - IC  6 QC 14.2 mg/kg - IC  6 QC 28.4 mg/kg – IC  3 QC 1.13 mg/kg (extracts at +4°C for 48h) - IC  3 QC 2.84 mg/kg (extracts at +4°C for 48h) - IC  3 QC 14.2 mg/kg (extracts at +4°C for 48h) - IC  3 QC 28.4 mg/kg (extracts at +4°C for 48h) - IC | Linearity,  Accuracy, precision,  Autosampler stability (IC) |
| Calibration (0.57, 1.13, 1.70; 2.27; 2.84, 5.7, 11.3, 22.7, 39.7, 56.7 mg/kg in IC)  3 QC 1.13 mg/kg (1 freeze/thaw cycle) - IC  3 QC 2.84 mg/kg (1 freeze/thaw cycle) - IC  3 QC 14.2 mg/kg (1 freeze/thaw cycle) - IC  3 QC 28.4 mg/kg (1 freeze/thaw cycle) - IC  3 QC 1.13 mg/kg (2 freeze/thaw cycles) - IC  3 QC 2.84 mg/kg (2 freeze/thaw cycles) - IC  3 QC 14.2 mg/kg (2 freeze/thaw cycles) - IC  3 QC 28.4 mg/kg (2 freeze/thaw cycles) - IC  3 QC 1.13 mg/kg (3 freeze/thaw cycles) - IC  3 QC 2.84 mg/kg (3 freeze/thaw cycles) - IC  3 QC 14.25 mg/kg (3 freeze/thaw cycles) - IC  3 QC 28.4 mg/kg (3 freeze/thaw cycles) – IC | Linearity,  Accuracy, precision,  freeze/thaw stability (IC) |
| Calibration (0.57, 1.13, 1.70; 2.27; 2.84, 5.7, 11.3, 22.7, 39.7, 56.7 mg/kg in IC)  3 QC 1.13 mg/kg (min.7 weeks, -70°C) - IC  3 QC 2.84 mg/kg (min.7 weeks, -70°C) - IC  3 QC 14.2 mg/kg (min.7 weeks, -70°C) - IC  3 QC 28.4 mg/kg (min.7 weeks, -70°C) - IC  3 QC 1.13 mg/kg (min.11 weeks, -70°C) - IC  3 QC 2.84 mg/kg (min.11 weeks, -70°C) - IC  3 QC 14.2 mg/kg (min.11 weeks, -70°C) - IC  3 QC 28.4 mg/kg (min.11 weeks, -70°C) - IC  3 QC 1.13 mg/kg (min.15 weeks, -70°C) - IC  3 QC 2.84 mg/kg (min.15 weeks, -70°C) - IC  3 QC 14.2 mg/kg (min.15 weeks, -70°C) - IC  3 QC 28.4 mg/kg (min.15 weeks, -70°C) - IC | Extended frozen storage  stability (IC) |
